# COVID-19 Outbreak Management and Vaccination Strategy in The United States of America

**DOI:** 10.3390/epidemiologia2030031

**Published:** 2021-09-10

**Authors:** Sara Aicha Amara, Estefany Daniella Díaz, Lakshmi Krishna Menon, Priyanka Singh, Liudmila Rozanova, Antoine Flahault

**Affiliations:** Global Health Institute, University of Geneva, 1211 Geneva, Switzerland; estefany.diaz@etu.unige.ch (E.D.D.); lakshmi.menon@etu.unige.ch (L.K.M.); priyanka.singh@etu.unige.ch (P.S.); antoine.flahault@unige.ch (A.F.)

**Keywords:** COVID-19, SARS-CoV-2, United States, vaccine, epidemiology, non-pharmaceutical interventions, economic impact, vaccine production, vaccine purchase, vaccine hesitancy, vaccine rollout, global health, public health

## Abstract

Four months after the first case of COVID-19 was reported in the United States, the SARS-CoV-2 virus had spread to more than 90% of all counties. Although the transmission of the virus can be grossly mitigated through non-pharmaceutical interventions and public health measures, risks of future outbreaks, emergence of more infectious variants, and disruptions to socio-economic life will probably remain until effective vaccines are administered to large portions of the global population. An exceptional collaboration between governments and the scientific community has led to the authorization of eight vaccines globally for full use, four of which were funded and developed in the United States. In this paper, we contextualize epidemiological, political, and economic impacts of the COVID-19 vaccination strategy in the United States of America between 20 January 2020, to 5 May 2021, with a key focus on vaccine hesitancy and public-private partnerships.

## 1. Introduction

The Coronavirus Disease-2019 (COVID-19), caused by Severe Acute Respiratory Syndrome Coronavirus 2 (SARS-CoV-2) escalated from Wuhan, China to a global scale in a matter of few months, directing the World Health Organization (WHO) to declare COVID-19 a pandemic on 11 March 2020. The United States of America (U.S. and U.S.A.) was one of the worst affected countries in the world; the virus spread across all 50 states and infected vulnerable populations, often through asymptomatic carriers [1]. The U.S. government had to adapt quickly, with non-pharmaceutical directives and economic measures to mitigate the effects of COVID-19 [2]. Several new COVID-19 variants were discovered in late 2020, posing a threat to the control of the disease.

As of 4 May 2021, there have been more than 32 million reported cases and 574,220 deaths [3]. With an accelerated funding into research and development for the COVID-19 vaccine and its rollout, a total of 312,509,575 vaccine doses had been distributed in the U.S., out of 44.5% of the population had received at least one dose of the vaccine, and 32% had been fully vaccinated [4].

In this study, we investigate vaccination strategy and rollout in the United States, between the first index case on 20 January 2020, to 5 May 2021. A comprehensive literature review was conducted with data and articles sourced from government websites, media and press releases, and reports from pharmaceutical companies in the private sector. This study is a part of a case series on investigations of COVID-19 vaccination strategies across countries.

The following sections will present an overview of U.S. geography, demography, politics, and economics, which will illustrate existing disparities within the healthcare system and the current epidemiological situation. This study will further examine the political management of the disease in the country, vaccine research, development and rollout, non-pharmaceutical interventions implemented, and public-private partnerships.

## 2. Case Presentation

### 2.1. Characteristics of the Country

The United States of America (U.S.) is a federal republic comprising 50 states and is home to 330 million people, the third-largest population in the world [5,6]. The U.S. mainland covers an area of 9.8 million square kilometers with a population density of 36.2 residents per square kilometer; 22.9% of this is population over 60 years old [7,8,9]. The U.S. climate varies across the landscape, with the average temperature ranging from 17 °C on the Pacific coast to −30 °C in arctic Alaska [10].

The nation has a federal structure of governance with powers split between federal (central) and state governments. The Federal Government is divided into three branches, each with separate powers as conferred by the Constitution: Legislative (Congress), Executive (led by the President), and Judicial (Federal courts) [11]. “All state governments are modeled after the federal government in terms of executive, legislative, and judicial branches” [12]. Although states have the primary responsibility for infectious disease outbreak, regulations established by the federal government’s pandemic response supports the notion that a successful battle against an outbreak, like the magnitude and intensity of COVID-19, necessitates a national response [13]. Nevertheless, public health laws and mandates differ from state to state, as with many aspects of National governance. The Center of Disease Control and Prevention (CDC) does not give any oversight to operations within any state. States enact public health measures as they deem fit, creating confusion, and in some cases—mistrust, among citizens [14].

**Figure 1:**
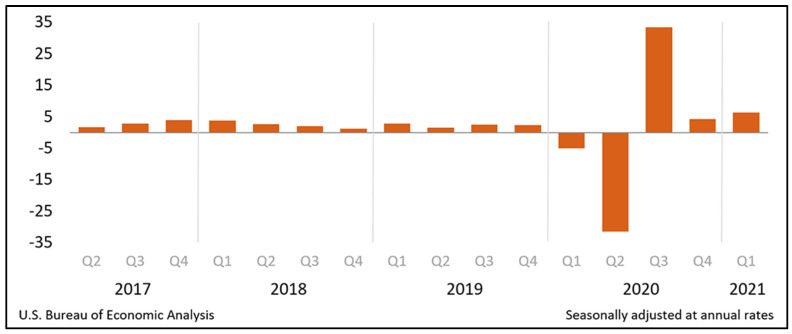
Real Gross Domestic Product (GDP): Percent change from preceding quarter. (Source: Reproduced from The United States (U.S.) Bureau of Economic Analysis) [17].

The U.S. is the world’s largest economy, earning more than 20 percent of the world’s total income [15] with an annual growth in GDP by 2.2% in 2019 [16]. The “Real Gross Domestic Product (GDP) increased at an annual rate of 6.4 percent in the first quarter of 2021” (Figure 1) [17]. The U.S. budget is a combination of all federal revenue and expenditures, where most of the revenue comes from taxes and borrowing from Treasury securities. Social Security insurance, military spending, and Medicare account for much of the spending [18]. The National Health Expenditure increased by 4.6 percent to reach $3.8 trillion USD in 2019 [19].

### 2.2. Epidemiological Situation

The epidemiological situation with regards to COVID-19 in the United States is presented in the following section, as based on open-access data from the Centers for Disease Prevention and Control (CDC), Johns Hopkins University (Centre for Systems Science and Engineering and Situation Reports), and the WHO.

**Figure 2:**
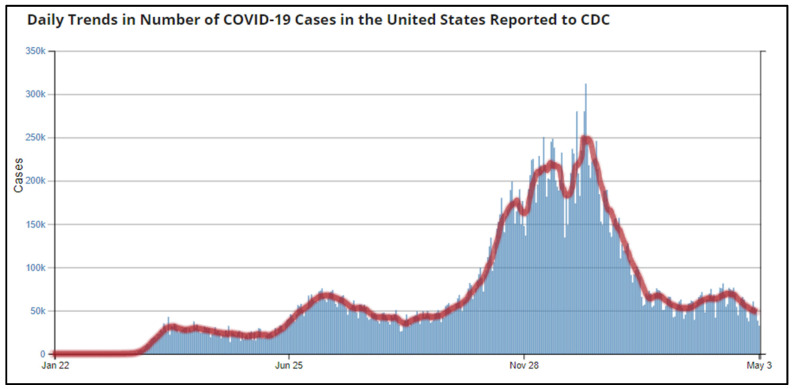
Daily Trends in Number of COVID-19 Cases in the United States Reported to Center for Disease Prevention and Control (CDC) (Source: Reproduced from the CDC) [4].

On 31 December 2019, China announced a cluster of pneumonia cases in people at Wuhan, Hubei Province [20]. The U.S. recorded its first index case of COVID-19 on 20 January 2020; a 35-year-old man presented with a four-day history of cough and fever from Snohomish County, Washington [21]. With upwards of 9800 cases of infection reported worldwide and more than 200 deaths, the WHO declared the novel coronavirus as a Public Health Emergency of International Concern on 30 January 2020 [22,23]. On 31 March 2020, CDC recorded 140,000 positive cases and 2000 deaths in the U.S. [24]. By January 2021, the nation reported its single highest number of new COVID-19 cases: 299,904 infections and 3844 deaths [4] (Figure 2).

**Figure 3:**
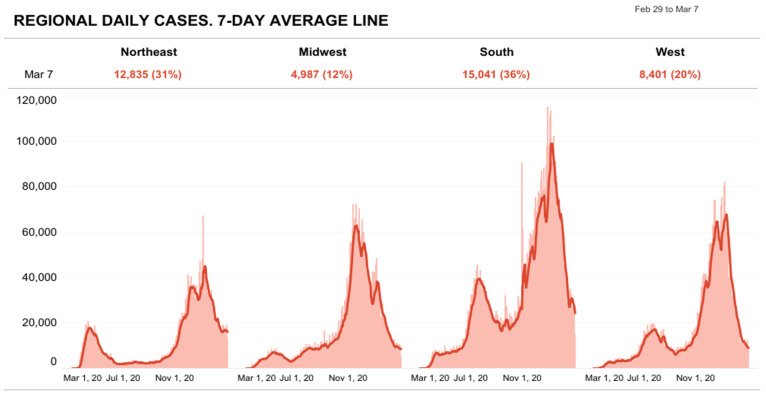
Counties Classed as U.S. Regions: Regional daily cases as 7-day average line” (Source: Reproduced from The COVID Tracking Project) [25].

All states experienced a surge of infection, and more commonly in autumn and winter [25]. In March 2020, the northeast coast saw 56 new cases per 100,000 residents. In autumn, there were up to 3000 new cases per 100,000 residents every month in other midwestern states. These numbers decreased significantly in February 2021, with fewer than 2000 cases per 100,000 residents, and are illustrated in Figure 3 [26].

**Figure 4:**
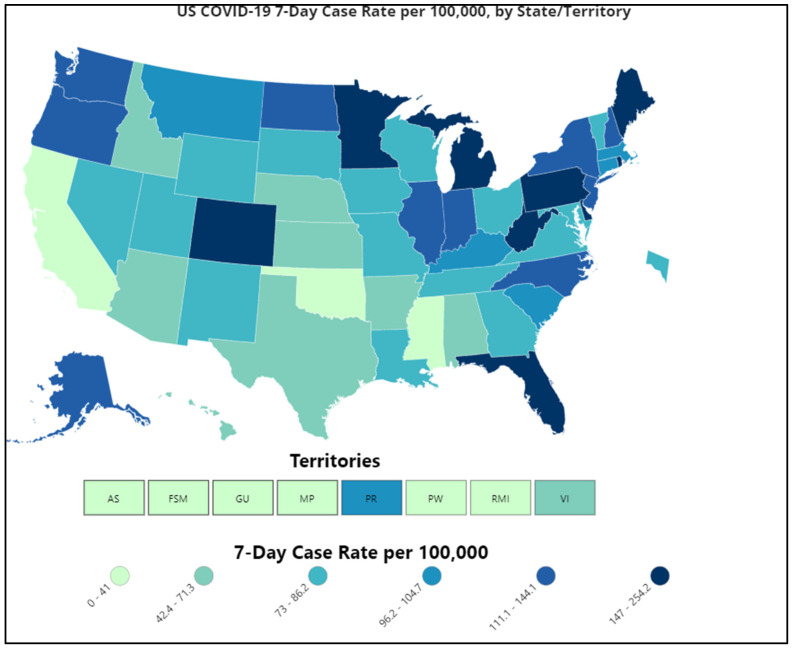
COVID-19 Case Rate in the U.S. Reported to the CDC, by State/Territory (cases per 100,000) (Source: Reproduced from the CDC) [4].

As of 5 May 2021, the country has recorded 32,491,117 infections and 577,329 deaths [26]. Of the total reported cases across the United States, Michigan, Minnesota, Maine, Texas, and Florida had the highest numbers of infections (Figure 4) [4]. Furthermore, the transmission of SARS-CoV-2 variants, with the detection of first variant of concern (VOC), B.1.1.7, in January 2021 in 12 U.S. states, has caused catastrophic problems in counties with low vaccination rates. On 19 April 2021, the U.S. Department of State released a Travel Advisory Update recommending against travel to 80% of countries worldwide [27]. As of 10 April 2021, there have been 20,915 confirmed cases of the B.1.1.7 variant.

Improved sanitary measures, coupled with high vaccination rates, have subsided COVID-19 cases, including the most recent outbreaks in Michigan, Minnesota, and Illinois [3]. By 30 April 2021, the CDC recorded an average of 626 deaths per day; this is the lowest daily mortality average since 27 June 2020 (508 deaths per day). National test positivity is also declining, dropping from 5.43% on 12 April 2021, to 4.47% on 27 April 2021 [28].

### 2.3. Immigration Crisis

The protracted immigration crisis of asylum seekers and unaccompanied minors from shared borders with Mexico and Canada has been disrupted due to the pandemic. Tens of thousands of people are confined to detention centers, despite high risks of viral transmission. These restrictions were put in place to prevent “the introduction of [a communicable] disease into the United States” [29]. Additionally, immigrants are “disproportionately represented in some of the most important services during a pandemic, such as health care, elder care, and food security” [29]. They also lack access to safety-net services, and are less likely to have healthcare coverage or meaningful incomes [29].

**Figure 5:**
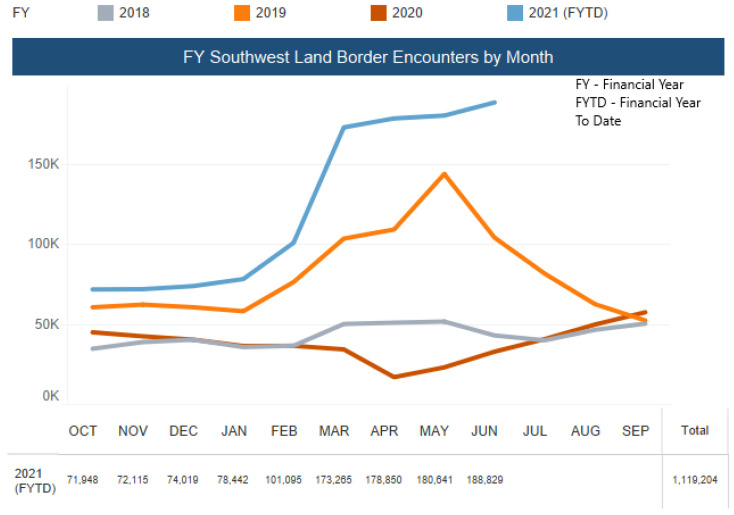
Encounters of Unaccompanied Minors by the U.S. Border Patrol along the South-West Border (Source: Reproduced from the U.S. Customs and Border Protection) [33].

On 27 March 2020, the USD 2 trillion COVID-19 aid package approved by the Senate omitted economic aid and access to testing and healthcare for immigrants [30]. In fact, the U.S. Customs and Border Protection (CBP) encountered more than 18,000 unaccompanied minors in March 2021 (Figure 5) [31], which is slowly developing into a humanitarian crisis as it “contributes to increased risk for COVID-19 outbreaks in these congregate settings” [32].

### 2.4. Racial Disparities

**Figure 6:**
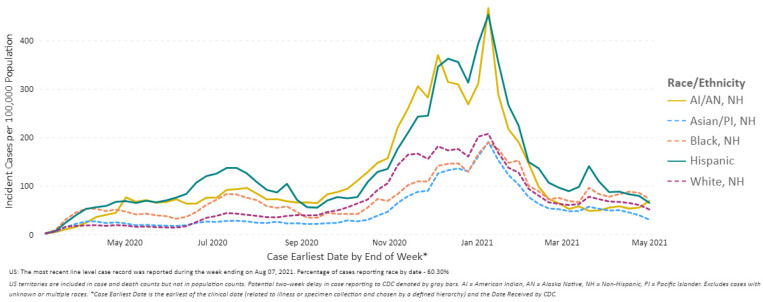
COVID-19 Weekly Cases per 100,000 Population by Race/Ethnicity, United States between 1 March 2020 and 1 May 2021 (Source: Reproduced from the CDC) [4].

COVID-19 disproportionately affected people from different racial and ethnic minority communities in the United States, as evidenced in the increased risk of infection, hospitalization, and death [34]. According to Blumenthal et al., 20% of all COVID-19 cases and more than 22% of COVID-19 related deaths, were among Black communities, who comprise 13% of the U.S. population [35]. Hispanics, who are 18% of the U.S. population, account for a third of all new COVID-19 cases in the United States (Figure 6) [36].

**Figure 7:**
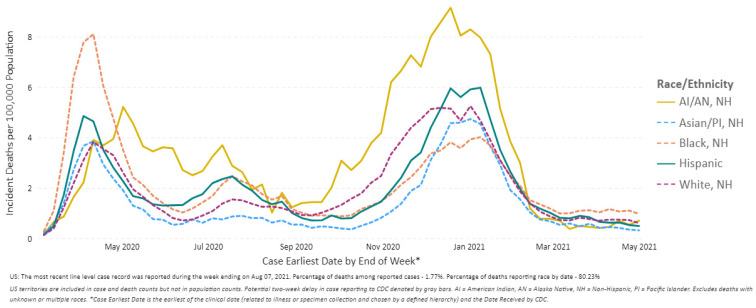
COVID-19 Weekly Deaths per 100,000 Population by Race/Ethnicity, United States between 1 March 2020 and 1 May 2021 (Source: Reproduced from the CDC) [4].

In the unique case of COVID-19, lack of comprehensive health coverage and employment in precarious industries continues to result in reduced access to treatment, and a higher prevalence of comorbidities; this makes populations more susceptible to infection [37]. When infected, racial minorities are more likely to seek treatment in safety-net facilities (public hospitals and primary healthcare facilities) that are already overburdened by acute-care demand [35]. A noticeable number of Black communities suffered more incident deaths due to COVID-19 during the early phase of the pandemic (Figure 7). COVID-19 transmission in U.S. counties with fewer Black communities is correlated to lower deaths. This points to socio-demographic factors that influence geographical distribution. Several studies have shown that Black and Hispanic workers generally represent the “essential workforce”, putting them at higher risk of infection [38,39], in addition to pre-existing health disparities [40]. These communities are also getting vaccinated at disproportionately lower rates, with barriers including language, onerous proof of eligibility, availability of transportation to vaccination centers, or internet access. In a longitudinal study, Lawton et al., 2021, conclude that an equitable and accessible health system is required to care for vulnerable populations in future pandemics [41].

### 2.5. United States’ Healthcare System

**Figure 8:**
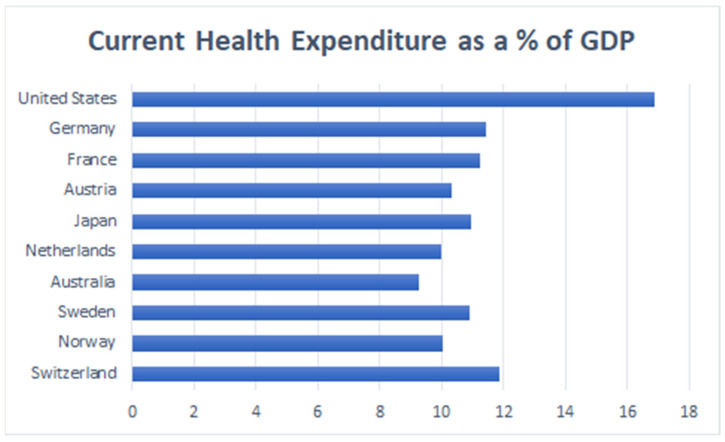
Current Health Expenditures as percentage of GDP, 2018. (Source: Adapted from the World Bank Database) [44].

Although the U.S. had a high healthcare spending of 17.7% as a percentage of GDP in 2018 [42], it’s healthcare system is fragmented through disconnected public-private insurance systems, federal, state, and local governments, and other institutions and individual providers (Figure 8) [43].

**Figure 9:**
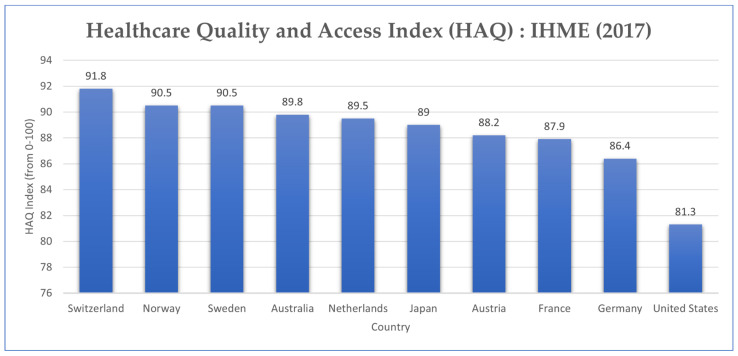
Healthcare Quality and Access (HAQ) Index Rating, 2015 (Source: Adapted from Our World in Data) [46].

The 2010 Affordable Care Act (ACA) provides subsidies for lower-income households. However, 26.1 million people did not have health insurance in 2019 [45]. In a measure of healthcare coverage and quality index amongst other OECD countries, the United States ranks last, suggesting higher rates of preventable mortality than peer countries (Figure 9) [46].

Emergency physician Dr. Maia Dorsett remarked, “COVID-19 is a slow-moving mass casualty incident (MCI), with the number and scale of casualties exceeding the available resources, such as staff and equipment” [47]. Hospitals in the U.S. have been rewarded for delivering elective surgical procedures to well-insured patients while dis-incentivizing them for providing the most necessary and urgent care. During the pandemic however, hospital profits have plunged in response to cancellations of elective procedures [48]. Between May 2020 and June 2020, 266 hospitals across states have furloughed their staff due to this decline in operating revenue [49]. Rises in unemployment or cross-cutting measures affect access to employer-sponsored health insurance for many households.

According to the Commonwealth Fund, 31 million people were uninsured and over 40 million were believed to be underinsured before the pandemic [35]. This points to lingering disparities in the health insurance system, which were exacerbated by the federal government’s withdrawal of general subsidies for the ACA, as well as the fact that undocumented immigrants were ineligible for subsidized coverage [22].Telemedicine was the main driver of the transition of the healthcare system during the COVID-19 pandemic. At the epicenter of the pandemic, New York hospitals witnessed an increase in virtual visits from 102.4 daily visits to 801.6 daily visits (683% increase) between the age groups of 20–44 years [23].

## 3. Management and Outcome

### 3.1. Political Management of the COVID-19 Outbreak

The U.S. was struck by the COVID-19 pandemic during their 2020 presidential election, which shaped the Republican and Democratic campaigns [50]. Data collected via the Gallup Panel as part of its COVID-19 tracking poll show approximately 25% of Republican respondents are “worried about getting the coronavirus,” whereas this percentage increases to almost 80% among Democratic respondents [51]. Similarly, while over 55% of those who identify as Republican are “ready to return to normal activities”, only 5% of Democratic respondents echo this sentiment.

President Donald Trump declared the novel coronavirus a national emergency on 13 March 2020, thereby unlocking billions of dollars in federal funding to control the outbreak in the U.S. [52]. By 26 March 2020, the Senate passed the Coronavirus Aid, Relief and Economic Security (CARES) Act, which provided USD 2 trillion in aid to hospitals, businesses, and state governments. This amounts to nine percent of the total Gross Domestic Product (GDP), making it the largest economic stimulus package in U.S. history.

More impressively, these investments were made before preliminary reports on the efficacy of the vaccine; federal regulators granted Emergency Use Authorization (EUA) to Pfizer-BioNTech and Moderna in December 2020 [53]. Operation Warp Speed (OWS) remains the single most effective mechanism that coordinated vaccine research and development among private companies and U.S. government bodies, including the Department of Defense, Department of Health and Human Services (HHS), the Food and Drug Administration (FDA), and the Center for Disease Prevention and Control (CDC) [54]. The biggest threat to OWS’s success is the reluctance of people to receive safe and recommended available vaccines, known as “vaccine hesitancy.” Nationwide, there continues to be a decrease in the number of new doses administered [55].

### 3.2. Challenges to COVID-19 Vaccine Acceptance

In a study of 1878 U.S. residents, 22% of respondents reported being hesitant to take vaccines should they be available to them [56]. Vaccine hesitancy was influenced by sociodemographic factors such as race, gender, employment, and place of residence. Other predictors include a historical mistrust with health care, lower levels of awareness, pre-existing vaccine hesitancy, and politicization of COVID-19.

There is a distinction to be made between vaccine hesitancy and vaccine refusal. In one study, 15% of persons who said they were at least somewhat supportive of vaccines said they would not get a COVID-19 vaccine [57]. Uncertain respondents may be waiting for more information about safety and efficacy in clinical trials, and how their social networks react [56]. Concerns were also raised that the vaccine development process is being rushed for political ends, and with regards to the inconsistencies in Non-Pharmaceutical Interventions (NPIs) have been implemented both between, and within, political parties [58]. Other logistic failures to mitigate COVID-19, including personal protective equipment shortages, and frequently changing guidelines between and within political parties regarding NPIs have also further eroded the public’s trust in government response [59].

In response, the CDC has developed a framework on building confidence in COVID-19 vaccines, which includes guidelines such as engaging in discussions where personnel at different levels can provide input and ask questions, building trust by communicating both what is known and not known, and collaborating with local partners [60]. Recently elected President Joe Biden has also dedicated up to USD 3 Billion to a nationwide vaccine promotion campaign [61]. This will benefit populations impacted by disparities in geographic regions or economic strata, and fund initiatives such as culturally competent door-to-door outreach, engage community leaders, or provide transportation to vaccination sites [61]. While OWS has been an unparalleled effort to rapidly bring to market safe and effective vaccines, a similar program is needed to promote acceptance of those vaccines.

### 3.3. Non-Pharmaceutical Intervention Measures Undertaken by Health Authorities

Non-pharmaceutical interventions (NPIs), such as physical distancing, PPE, hand hygiene, and country-wide lockdowns, have been at the forefront of outbreak control during the COVID-19 pandemic. A national plan was released in April 2020, stating that until a vaccine was available, current measures should rely on “traditional public health methods,” including contact tracing [62]. The U.S. adopted the “Test, Trace, Isolate” guideline (a proposal created by WHO) in order to mitigate the spread of the new coronavirus in its population [63]. According to Grantz, et al., this method contributes to the reduction of the effective reproductive number (Re), which is defined by the number of people who can be infected by a person that has contracted COVID-19 [64].

The “Test, Trace, Isolate” strategy was under the instruction of the CDC, who provided guidance, resources, and support to the health departments across the United States. The use of digital tools or contact tracing mobile applications was promoted to notify cases of infection, as well as to alert the subjects with whom there was close contact (anyone who has been within 6 feet of the infected person for a longer time or equal to 15 min over a 24 h period) [65], to subsequently be monitored and tested for free, with the aim of covering and eradicating the virus even in the most vulnerable populations [66].

In June 2020, the CDC called for at least 100,000 contact tracers to address a surge of cases. At the start of the pandemic, the U.S. had 2200 contact tracers, though this number has now increased to 50,000, with wide variations among states. One study showed that even with the implementation of this strategy, more than half of COVID-19 positive cases did not provide their contact details; while an average of 17 people per case were identified in Taiwan, 2 in the United Kingdom, and 1.4 in France, some states in the U.S. have traced zero, or less than one person per positive case [67].

In regard to other non-pharmaceutical interventions, mask guidelines have evolved over the course of the pandemic. On 15 March 2020, the CDC made no mention of masks when it recommended that any gathering in the United States be limited to 50 people. This changed in April 2020, when all U.S. residents were encouraged to wear a mask outside their homes to complement other NPIs, including physical distancing and handwashing [68].

On 27 April 2021, the CDC issued a new and carefully written mask mandate: “Americans who are fully vaccinated against the coronavirus no longer need to wear a mask outdoors while walking, running, hiking or biking alone, or when in small gatherings, including with members of their own households. Masks are still necessary in crowded outdoor venues like sports stadiums” [69]. However, it remains largely up to state officials to determine what restrictions, if any, to impose to slow transmission. In the following section, we make a comparison between infection control and prevention measures in California, Michigan, New York, and Texas (Table 1).

California was one of the first states to issue stay-at-home directives, which continue to date [71]. On 28 August 2020, the state released the Blueprint for Safer Economy for a gradual reopening of businesses [71]. If vaccine supply is sufficient for Californians 16 years of age or older and hospitalizations are stable and low, California aims to reopen its economy on 15 June 2021. Michigan was the state that, at the time this manuscript was written, saw the most recent COVID-19 outbreak on 13 April 2021. This new wave of infections primarily affected younger persons, due to school-affiliated extra-curricular activities in the spring break [72]. Since Michigan saw a 48% decline in weekly reported cases. New York has reported one of the highest numbers of deaths per 100,000 inhabitants, at 239 [73]. Neighborhoods with lower incomes and a greater number of people living together had higher case fatality rates [74]. Texas lifted the mask mandate and capacity restrictions on all businesses on 10 March 2021, and cases have not surged since [75]. This can be attributed to vaccinations, which have climbed steadily as the federal government cleared all people 16 and older eligible for the COVID-19 vaccine in every state [76].

### 3.4. Economic Effects of COVID-19

The pandemic’s economic impact is the difference between what is expected (based on historical trends), and what actually happens at the given period of time. Globally, a correlation is seen with decreasing deaths per million people and negative impact on GDP growth [77]. The decline in the employment-to-population ratio in the United States in April 2020 was 51.5%, while the historical trend predicted 61.3% [78]. In other words, there were fewer people employed than what was expected before the pandemic. In comparison, excess deaths had a weak correlation to employment displacement. The State of New York, which had the highest number of excess deaths (55,600, including 31,500 from New York City alone) between 15 March 2020 and 13 March 2021 [79], experienced a modest decline in employment-to-population ratio [80].

On 25 March 2021, the Bureau of Economic Analysis (BEA) reported real gross domestic product (GDP) decreased at an annual rate of 5.0% in the first quarter of 2020 and 31.4% in the second quarter of 2020; this is the largest quarterly decline of GDP on record [81]. While the GDP rose at an annual rate of 33.1% in the third quarter of 2020, it remains 2.9% below pre-pandemic levels [82]. A sharp decline followed by rebound in personal consumption expenditures drove both the decline and partial recovery of real GDP in 2020 [82]. The nature of anti-COVID-19 measures, including stringent lockdowns and stay-at-home orders, changed certain consumer behaviors, such as spending on nondurable goods including gasoline, or new clothing.

The Trump administration provided USD 2.2 Trillion in funds for individuals, small businesses, large corporations, and local governments, through the Coronavirus Aid, Relief and Economic Security (CARES) Act signed on 26 March 2020 [83]. Individual relief included $1200 cash payments for persons eligible based on their 2019 income tax return, and unemployment payments of $600/week for a period of four months [83]. In response to the crisis, the Federal Reserve lowered the inter-bank lending rates, reduced short-term interest rates, and created new emergency credit facilities [84].

Going forward, the economy may fully recover, but there could be longer-lasting impacts for those whose industries that have been hit the hardest by the pandemic (construction, entertainment, and recreation), individuals from geographical areas that suffered large losses (of human capital and businesses), or low-income households without the possibility of telework [84,85].

## 4. Vaccination Strategy

### 4.1. Vaccine Characteristics and Registration Procedure

As of May 2021, FDA’s Center for Biologics Evaluation and Research (CBER) have authorized three COVID-19 vaccines for emergency use in the U.S.: Pfizer-BioNtech, Moderna, and Janssen/Johnson & Johnson [86]. Under normal circumstances, patients have access to a vaccine only when the FDA licenses its use on the market under a Biologics License Application (BLA) or allows its use in clinical trials under an investigational new drug (IND) application. However, in emergency situations, like the COVID-19 pandemic, the vaccine can be made available to interstate commerce under an Emergency Use Authorization (EUA) prior to the availability of complete long-term safety data [87,88].

EUAs are issued by the FDA to facilitate the supply of unapproved medical drugs upon satisfying four statutory criteria:

1. The CBRN agent to which the EUA applies can endanger life, or cause different conditions or diseases [87].
2. Reasonable evidence to believe that the medical product “may be effective” in preventing, diagnosing, or treating the disease or condition [87].
3. The product’s identified and prospective benefits exceed its identified and prospective risks [87].
4. There are no other suitable alternatives available or approved for the prevention, diagnosis, or treatment of the disease or condition [87].

Before supporting the issuance of an EUA by FDA, the U.S. Department of Health and Human Services (HHS) must ensure that current events support the authorization by satisfying at least one of the four determinations from the HHS emergency declaration. This entails for the HHS Secretary to conclude that a public health emergency exists that threatens, or may threaten, national security or the health and safety of U.S. citizens living abroad, and involves a chemical, biological, radiological or nuclear (CBRN) agent [88].

In 2020, three major companies—Pfizer, Inc., ModernaTX, Inc., and Janssen Biotech, Inc.—conducted multi-phase, blinded, randomized, placebo-controlled studies on the safety, immunogenicity, and efficacy of the COVID-19 vaccines [89]. During the fourth quarter of the same year, the petitions that were submitted included, by FDA requirement, a data sheet for the recipients and vaccination providers describing the vaccine, such as the name or intended use, description of the targeted disease, dosage, as well as elements to discuss with a medical provider in case of adverse reactions [90,91]. The characteristics of COVID-19 Vaccines authorized for emergency use are summarized in Table 2.

#### 4.1.1. Pfizer-BioNTech

On 20 November 2020, Pfizer, Inc., under the name of Pfizer-BioNTech, submitted an EUA for the BNT162b2 COVID-19 vaccine, intended for individuals ≥16 years of age. Based on nucleoside-modified messenger RNA (modRNA) technology, the vaccine encodes the viral spike glycoprotein of SARS-CoV-2 triggering an immune response [90]. Data on efficacy from ongoing clinical trials supported a 95% (95% credible interval 90.3, 97.6) efficacy on the vaccine’s ability to prevent COVID-19. Anupdated study confirmed that efficacy remained for at least six months after the second dose [90,91]. This study (C4591001) was conducted in the United States, Argentina, Brazil, Germany, South Africa, and Turkey. Throughout Phases 1, 2, and 3, it involved a total of 43,693 subjects, organized in two main groups: 21,847 in the BNT162b2 (vaccine) group, and 21,834 in the placebo (saline solution) group (Table 3) [88,91]. On 7 May 2021, Pfizer and BioNTech announced the submission of a BLA to the FDA “with the goal of securing full regulatory approval of the vaccine in the coming months.” [93]. The companies will report data incrementally, as the BLA requires long-term follow-up [93]. Prior to this announcement, Pfizer and BioNTech also submitted an application to extend the EUA to children and adolescents aged 12–15 years, after sharing positive results from a study conducted in this age group [94].

#### 4.1.2. Moderna

On 30 November 2020, 10 days after Pfizer-BioNTech’s request, ModernaTX became the second company to seek FDA approval of their Moderna mRNA-1273 COVID-19 vaccine, intended for individuals 18 years of age or above. [96]. Like the Pfizer-BioNtech vaccine, it is a modRNA investigational vaccine that has not been licensed for any indication. Evidence from clinical trials on efficacy suggests that the vaccine is 94.1% effective at preventing COVID-19 [97]. The efficacy study (P-301) was conducted in 99 locations across the United States. Phase 3, whose objective was to prove vaccine efficacy, involved a total of 30,351 individuals: 15,181 in the mRNA-1273 group, and 15,170 in the placebo group (Table 4) [97]. In a report published in early May 2021, Moderna expressed its intention to initiate a BLA in the U.S. for full approval of its vaccine in the coming weeks. Recent progress in a clinical trial for adolescents aged 12–17 years revealed 96% efficacy in the prevention of COVID-19, with no significant safety concerns to date [98].

#### 4.1.3. Janssen Biotech, Inc.

Janssen Biotech Inc., was the third sponsor submitting the EUA’s request to the FDA approval on the Ad26.COV2.S COVID-19 vaccine, also intended for individuals 18 years of age or above. The vaccine contains a recombinant, replication-incompetent human adenovirus serotype 26 (Ad26) vector, encoding the viral spike (S) glycoprotein of the SARS-CoV-2. Analysis of the efficacy data showed that the vaccine is 66.9% effective (95% confidence interval (CI): 59.0, 73.4) in preventing COVID-19 [99]. Unlike the two previously mentioned vaccines, Ad26.COV2.S had two Phase 3 efficacy and safety studies: 3001 (1-dose regimen) and 3009 (2-dose regimen). However, only 3001 was considered for EUA approval. This study was developed in the United States, Brazil, South Africa, Argentina, Chile, Colombia, Mexico, and Peru, and involved 43,783 individuals: 21,895 in the Ad26.COV2.S group, and 21,888 in the placebo group (Table 5) [100]. Later in 2021, the company intends to submit a BLA to the FDA, as more data are collected [101], and has enrolled adolescents aged 12 to 17 years in an ongoing Phase 2a clinical trial in an effort to expand vaccine coverage [102].

### 4.2. Production and Purchase Schedule for Vaccines and Related Medical Products

In an initial contract signed in July 2020, the U.S. government agreed on a 100 million dose purchase with Pfizer and BioNTech, for a total of $1.95 billion. The U.S. has the option to acquire up to 500 million additional doses under this agreement [103]. A second contract was signed in December 2020 and a third in February 2021, each for an additional 100 million doses of the Pfizer-BioNTech vaccine for $1.95 billion, bringing the total doses of vaccine scheduled for distribution in the United States up to 300 million by July 2021 [104]. Since these contracts are under the purview of the federal governments, all vaccines are administered free of charge to the U.S. population [105].

Moderna, meanwhile, has increased its shipping capacity fivefold and is expected to deliver about 50 million doses per month to the United States, in order to meet the agreed-upon 300 million doses by July 2021. By the end of 2020, the company was able to deliver 17 million doses, and 117 million by April 2021. An additional 100 million doses are to be delivered by the end of May 2021, and another 100 million doses by July 2021 [106,107]. The contract signed with Moderna is worth $4.94 billion in total [108]. As for Johnson & Johnson, the company expects to deliver 100 million doses to the U.S. government during the first half of 2021 for a total of $1 billion [109]. Publicly disclosed contracts for the purchase of vaccines and ancillary products are reproduced in Table 6 and Table 7 from the Congressional Research Service (CRS) Insight IN11560 [108,110,111].

### 4.3. Prioritization of Target Groups

As a fundamental component of “Values to Priority Groups”, a vaccination strategy WHO’s Strategic Advisory Group of Experts on Immunization (SAGE) developed, is a framework of 6 principles: Human Well-Being, Equal Respect, Global Equity, National Equity, Reciprocity, and Legitimacy. These outline which populations, including the elderly, first-responders, health personnel, and individuals with comorbidities, should be prioritized in order to achieve a coordinated and effective vaccination response [112].

The Advisory Committee on Immunization Practices (ACIP) is a 15-member panel of specialists responsible for providing directives regarding the use and administration of vaccines in the United States [113]. In December 2020, they issued directives for individuals to be vaccinated in accordance with age, risk for prolonged exposure in healthcare settings, and pre-existing medical conditions [114].

The recommendations from WHO, ACIP, and CDC were approved and published as official guidelines for the vaccine rollout in the United States [94].

#### 4.3.1. Phase 1a

Phase 1a corresponds to two groups that account for nearly 24 million people. It focuses primarily on healthcare personnel (HCP), as their work and occupational settings are hotspots of high risk of SARS-CoV-2 exposure. According to Banfyopadhyay et al., nearly 20% of COVID-19 patients were identified as healthcare workers [115]. During the second quarter of 2020, an average of 1.7 deaths per 10,000 health care workers was reported [116]. On the other hand, the second group of phase 1a corresponds to residents from long-term care facilities (LTCF) who, due to their age, communal living situation, and the high prevalence of subjacent medical conditions, are highly susceptible to contracting and developing acute COVID-19 disease [117]. Although this group represents only 4% of cases of COVID-19 infection, deaths exceed this percentage by a factor of 8, which means that more than 30% of deaths in the United States have occurred in individuals residing in these areas [118].

#### 4.3.2. Phase 1b

Phase 1b of the vaccination program is directed to approximately 15% of the total U.S. population (nearly 50 million) [119]. It includes a broader spectrum of personnel who, while not working in healthcare settings, are key for the functioning and operation of the country’s infrastructure and services. Essential workers are often expected to work in close contact with other individuals, and can be “first responders, corrections officers, food and agricultural workers, U.S. Postal Service workers, manufacturing workers, grocery store workers, public transit workers, those who work in the education sector, as well as child care workers” [119]. The second group of phase 1b is constituted by individuals older or equal to 75 years old, who possess a high risk of severe effects or death due to COVID-19 infection [119].

#### 4.3.3. Phase 1c

Phase 1c covers approximately 129 million inhabitants, including people between 65 and 74 years of age who don’t reside in LTCF but may share a high risk of hospitalization, intubation, or death due to coronavirus infection [119]. Furthermore, it targets individuals between 16 and 64 years of age with pre-existing conditions, and essential workers who were not eligible for inclusion in the previous phase, and who operate in one of the following areas: “transportation and logistics, water and wastewater, food service, shelter and housing, finance, information technology and communications, energy, legal, media, public safety, and public health workers” [120]. According to the CDC, only 10% of people hospitalized for COVID-19 are reported to have no pre-existing or underlying medical condition. The remaining 90% generally present hypertension, obesity, or metabolic and/or cardiovascular diseases [121].

On 4 May 2021, President Joe Biden declared a goal of immunizing 70% of the U.S. adult population with at least one COVID-19 shot by 4 July of the same year. This target means administrating approximately 100 million doses during May and June 2021 [122].

#### 4.3.4. Phase 2

Once the three stages of phase 1 have been completed, phase 2 proceeds with populations under 18 years of age, or those who were not qualified to belong in phase 1. Since clinical trials in children and adolescents are underway, it is not yet possible to determine a precise phase of vaccination for adolescents [119].

### 4.4. Vaccine Supply Chain and Dynamics

CDC recommended administering the COVID-19 vaccine to healthcare workers and residents of long-term care facilities. While each state can define the priority group to which it allocates vaccines after these priority-groups, recommendations have first been extended to teaching staff and childcare workers, and then extended to all U.S. citizens on 1 May 2021 [123].

COVID-19 vaccines are allocated to jurisdictions “based on the number of people 18 years or older in the jurisdiction in proportion to the entire U.S. population”. Jurisdictions select the entity to which they deliver the vaccines (retail pharmacies, hospitals, or health departments). Orders of vaccines are placed through the CDC’s platform Vaccine Tracking System (VTrckS) [124]. The VTrckS is a technology tool for managing the entire supply chain of publicly funded vaccines, from purchasing to distribution [125].

Given that COVID-19 vaccines have specific temperature storage requirements (Pfizer-BioNTech: −70 °C, Moderna: −20 °C, Janssen/Johnson & Johnson: 2–8 °C) [126,127], in order to reduce potential breaks in the cold chain and ensure feasibility of supply and safety of their recipients, the CDC recommends performing periodic inspections of the storage unit, correcting vaccine displacements, recording the daily minimum and maximum temperatures, and removing expired vaccines [128].

**Figure 10:**
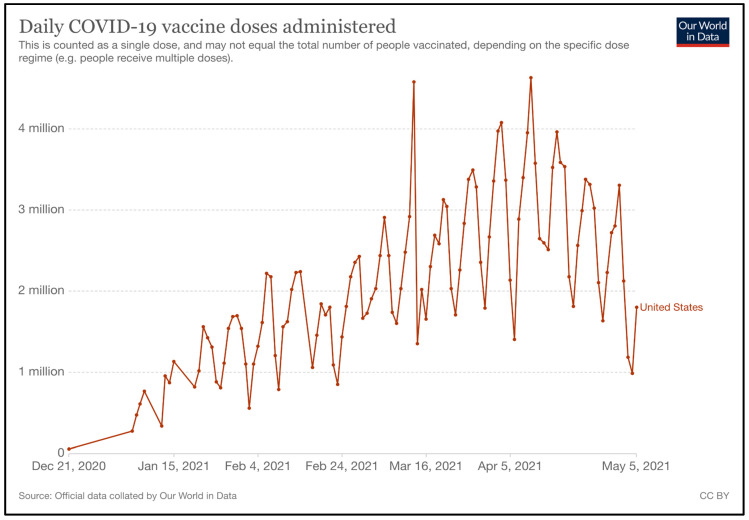
Daily COVID-19 Vaccine Doses Administered (Source: Our World in Data) [130].

Vaccines are distributed in a centralized manner to allow the government to have full control on vaccine uptake. McKesson company, who was previously responsible for the distribution of the H1N1 vaccine in the U.S., is currently in charge of the distribution of Moderna and Johnson and Johnson’s ancillary supply kits. This is within the scope of their contract with the CDC, with regards to thedistribution of vaccines during pandemics [129]. For Pfizer-BioNTech, although McKesson distributes their ancillary supply kits, the company is not responsible for distributing the vaccine due to the vaccine’s ultra-cold storage requirements [127]. Pfizer has instead adopted a “just-in-time” system, which ships its frozen vials directly from the Kalamazoo (Michigan) or Pleasant Prairie (Wisconsin) plants to point-of-care within two days [112]. The daily COVID-19 vaccine doses administered are illustrated in Figure 10 [130].

### 4.5. Clinical Management of Vaccination

As part of the statements regarding vaccination and the spread of SARS-CoV-2, the CDC has issued a series of recommended guidelines for non-healthcare individuals who have been completely vaccinated. These guidelines were in effect until the emergence of new evidence on variant B.1.617.2 (Delta), and recommendations regarding indoor masking for fully vaccinated people have been subject to change [131].

After two weeks of receiving both doses of Pfizer-BioNTech or Moderna vaccine, or the single dose of Janssen/Johnson & Johnson vaccine, people are allowed to stop wearing masks or physically distancing in closed areas with other vaccinated individuals, or unvaccinated individuals who are not at high risk of acute COVID-19 disease. For all other events in enclosed environments, the use of masks by both parties is still required [131]. Open-air activities and sports can be resumed without the mandatory use of masks, except for large events where transmission at the community level is high, and where the unvaccinated population outweighs those vaccinated [131].

Those vaccinated will not have to comply with quarantine or be tested before or after the arrival at the final location, if traveling within the United States [132]. For all international travel, it remains mandatory to adhere to the COVID-19 regulations of destination countries, regardless of vaccination status. However, quarantine is not required upon return to the United States for those fully vaccinated. It is also not imperative to be tested or placed in quarantine if asymptomatic after contact or exposure to another case of SARS-CoV-2 [105,131]. The above guidance applies to individuals who have been vaccinated with any of the FDA or WHO emergency authorized vaccines (Pfizer/BioNtech, Moderna, Janssen/Johnson & Johnson, AstraZeneca/Oxford, AstraZeneca-SK Bio, Serum Institute of India) [133].

Since the efficacy of COVID-19 vaccines in patients with chronic diseases or compromised immune systems continues to be studied, the CDC encourages adhering to their guidelines in consultation with healthcare providers. [105,134]. The CDC recommends vaccination regardless of previous SARS-CoV-2 infection. However, people with active SARS-CoV-2 infection are eligible for vaccination only after the end of the acute phase of infection, and when their isolation period is lifted. Authorized vaccines are not recommended for post-exposure prophylaxis, as they may not trigger an effective immune response within the short COVID-19 incubation period. Those with other underlying medical conditions are also eligible for COVID-19 vaccination in so far as there are no contraindications to any component of the vaccine (i.e., anaphylaxis or immediate allergic reaction of any severity) [92].

Health practitioners can request a consultation from the Clinical Immunization Safety Assessment COVIDvax program (CISA COVIDvax) for patients with specific underlying medical conditions unaddressed in CDC and ACIP guidelines [135]. A non-exhaustive list of underlying medical conditions eligible for vaccination may include immunocompromised people (HIV positive infection, or immunosuppressive treatments) and those with autoimmune diseases [92].

Preliminary findings have not suggested safety concerns for other populations, including pregnant and lactating people [136]. Data on the interchangeability of COVID-19 vaccines are also scarce; the safety and efficacy of administering two different vaccines continue to be evaluated. To date, the CDC recommends completing vaccination with the same product administered for the first dose [92].

To track the health status of individuals upon receipt of the first and/or second dose, CDC launched the voluntary-use mobile application *V-Safe*. This enables reporting of any post-vaccination symptoms. If the user accesses this application at the time of the first dose of the Pfizer-BioNTech or Moderna vaccine, it will issue a reminder for the second dose scheduled. Within the first 5 weeks, and up to 12 months after the last dose, the individual can answer check-in questionnaires, and report any changes [137].

The Vaccine Adverse Event Reporting System (VAERS), founded in 1990 by the CDC and FDA, is a national platform for passive monitoring of adverse events following vaccination; it comprises four components for vaccine safety monitoring [138]. Through this platform, healthcare workers and the general public complete an online form to declare any adverse post vaccination symptoms. The CDC and FDA may investigate these reports in case of unusual patterns [87,139].

### 4.6. Investigational Therapeutics for COVID-19

The two FDA-approved drugs, and others cleared for use under EUA, are being used to treat COVID-19 [140]. Although clinical management relies on symptomatic and supportive care, new therapeutics have been introduced for outpatient treatment in the early stages of the disease including, inter alia, and monoclonal antibodies. While there is no therapy that has shown to be clinically beneficial in the treatment of patients with mild to moderate symptoms, or those unlikely to develop severe disease presentations, anti-SARS-CoV-2 antibodies appear to have the highest clinical benefit when initiated as a treatment in the early stages of the disease [140,141]. Current recommendations for the clinical management of COVID-19 with monoclonal antibodies include bamlanivimab plus etesevimab or casirivimab plus imdevimab, both of which have been granted EUA when administered together [141].

Other popular treatments include Remdesevir, Dexamethasone, and Tocilizumab. Remdesevir is the only antiviral agent approved by the FDA for the treatment of COVID-19 and recommended for hospitalized patients requiring oxygen therapy [142,143,144,145]. Dexamethasone is a corticosteroid linked to improved survival and clinical benefit in mechanically ventilated patients [146,147,148,149]. Tocilizumab is an interleukin-6 receptor antagonist that, when combined with dexamethasone, improves survival in patients with rapid respiratory decompensation [150,151].

## 5. Discussion

The U.S. pandemic response is best characterized by its decentralized system of governance. While the national government has managed fiscal response by increasing funding for scientific research into testing vaccines, state governments have been responsible for non-pharmaceutical interventions, administering vaccinations, and re-opening economies. Though this expands speed and flexibility in vaccine rollout, it has also contributed to the heterogeneity of COVID-19 measures across the country. In this case study, we made a comparison between infection control and prevention measures in the following four states: California, which was one of the first states to issue stay-at-home directives; Michigan, which recorded an outbreak of cases at the time this manuscript was written; New York, which reports the one of the highest number of deaths per 100,000 inhabitants; and Texas, which was the first state to lift the mask mandate. These measures point to competing interests that exist between (a) the need to re-open the economy and return to normalcy, and (b) expend efforts to contain transmission and reduce health impacts. Future studies can systematically explore state-level data on COVID-19 metrics in relation to state-issued directives.

COVID-19 “exhibited clear geographic trends” in its transmission across the country [152]. This had impacts among racial and ethnic minorities and immigrant populations, particularly due to pre-existing inequities in social determinants of health, as well as state-level restrictions imposed to contain infection.

Although the “Test, Trace, Isolate” campaign was introduced as a public health recommendation to all individuals across the U.S., existing disparities such as the lack of insurance, unequal access to testing sites or health centers, and higher risks of viral exposure for minority groups and essential workers, meant that this program exacerbated gaps in the health system [153,154].

The first phase of vaccine rollout has been criticized for failing to address racial disparities within high-risk communities [155]. This phase prioritized healthcare workers andadults over the age of 65. Since White Americans are better represented in this demographic (76% of the U.S. senior population), less than 10 percent of those eligible for Phase 1 vaccinations were Black-Americans [156]. This is further challenged by barriers to vaccination that disproportionately affect racial minorities, who cite limited access to online services for COVID-19 vaccine registration, lack of transportation, and a general distrust in healthcare infrastructure [157,158]. Arace-conscious approach to vaccine deployment, coupled with sustained efforts to build trust in the health system, is essential to reducing the burden of the pandemic [155,157].

The economic impact of the COVID-19 crisis created a unique combination of demand shock, supply shock, and financial shock [152]. This was further complicated by the emergence of more infectious genetic variants of the original SARS-CoV 2, pointing to a need to invest in better surveillance systems that identify variants and trace transmission to facilitate effective public health responses. Today, the B.1.1.7 variant first reported in December 2020 is a primary concern in United States epidemiology [159].

To this regard, it is important to reiterate that our report focuses on the U.S. COVID-19 vaccination strategy up to 5 May 2021 SARS-CoV-2 variants of concern, which continue to be studied after this date, have not been addressed in depth. These include, the Beta Variant: B.1.351. 2, B.1.351.3, initially identified in South Africa, characterized by increasing virus transmission by 50%; Delta Variant: B.1.617.2, AY.1, AY.2, AY.3, initially identified in India, characterized by increasing virus transmission by up to 60%, and Gamma Variant: P.1, P.1.1, P.1.2, P.1.4, P.1.6, P.1.7, initially identified in Japan and Brazil, characterized by significantly reduce susceptibility to monoclonal antibody treatment) [160].

The United States has administered 74 doses per 100 persons, which is a stark contrast to the global rate of vaccination, which averages at 15 doses administered per 100 persons [161]. Despite a rapid vaccination response campaign, there continues to be discrepancies in progress across states, with “some having vaccinated a smaller share of their population with a first or single dose” and with more “vulnerable southern states lagging behind the national average” [55,161]. On 31 January 2021, OWS announced that the pharmaceutical companies that were granted EUAs have released 63.7 million doses, which is representative of only 32% of the 200 million doses that companies were expected to supply by the end of March 2021. Industrial scale production is challenged by tight production deadlines and restricted manufacturing capacities, as stated in a report issued by the Government Accountability Office (GAO) in 2021 [162].

Efforts to increase production capacity have been undertaken by supporting vaccine manufacturers to identify new manufacturing partners, and supervising construction projects to expand vaccine production capacity. Shortages in qualified staff are being addressed through a cooperation between OWS and the U.S. Department of State, to expedite visa clearance for engineers to oversee quality control of equipment produced overseas [162]. According to a McKinsey report, “current administration architecture and market demand are not keeping up with supply, and coverage will have to be nearly doubled by May to meet 80% coverage” [123].

The ACIP, as a part of the CDC, have developed a two-phase vaccination plan (Phase 1a, 1b, 1c, and Phase 2), which is guided by four key ethical principles: maximize benefits and minimize harms, mitigate health inequities, promote justice, and promote transparency [119]. This framework demonstrates an inclusive approach to all members of society by acknowledging their contributions to the community and health, education, or economic sectors, as well as acting as a mechanism to reduce health disparities.

Foreseeable challenges in the U.S. vaccination strategy are grounded in improving vaccine hesitancy and addressing vaccine inequities worldwide. In the words of Ursula von der Leyen, President of the European Commission, “no one is safe until everyone is safe” [163]. The United States pledged to provide the COVAX initiative, established by the World Health Organization and GAVI, the Vaccine Alliance, $2 billion USD to promote global equitable access to coronavirus vaccines in low- and middle-income countries [164]. On 5 May 2021, the United States made a landmark announcement that it supports waiving intellectual property and patent protections for COVID-19 vaccines, which is expected to improve access to vaccines for low- to middle-income countries [165]. However, there is considerable ambiguity in how logistical bottlenecks and trade agreements will be addressed.

Our review serves as a point of reference into how the United States initiated a national COVID-19 vaccination drive. We hope to support researchers in other scientific fields in the U.S. COVID-19 epidemiological situation between 20 January 2020, to 5 May 2021. As information is subject to change as the pandemic evolves, it is necessary to consider the limitations of our case report. First, given the non-immediate effect of vaccine deployment, drawing parallels between each vaccine phase and the COVID-19 transmission curve remains difficult to extrapolate. Second, data may have gaps in terms of how it was sourced, which assumptions were made, and how terms were defined. Therefore, our paper serves best as a scoping overview of subtopics as it relates to COVID-19 in the United States, and not as definite research. Third, the emergence of variants poses an increased risk to public health, and warrants global monitoring and research to inform the ongoing response to the COVID-19 pandemic. Fourth, mental health impacts of COVID-19, and debates on mandating vaccinations and/or testing continue to be concerns as the United States segues into reopening businesses and schools. These continue to unfold complexities that influence the chain of supply and demand, vaccine access and distribution, and vaccine hesitancy. More circumstantial and systematic research is required for each dimension of the United States’ COVID-19 vaccination response.

## Figures and Tables

**Table 1:** “NPIs and Case Counts in California, Michigan, New York, and Texas” [70].

	California	Michigan	New York	Texas
**Population**	39.5 M	9.9 M	19.5 M	29 M
**Cases**				
Total Reported	3,748,832	948,029	2,057,701	2,904,178
7-Day Average * + (14-Day-Change)	1766 (−25%)	3594 (−48%)	3148 (−46%)	3097 (−4%)
**Deaths**				
Total Reported	61,996	19,041	51,949	50,567
7-Day Average * + (14-Day-Change)	70 (−14%)	70 (+15%)	52 (−17%)	53 (−1%)
**NPIs**	Some businesses closed	Businesses mostly open	Businesses mostly open	Businesses open
	Masks mandatory indoors	Masks mandatory indoors	Masks mandatory indoors	Masks not mandatory
	Stay-at-home advisory			
**Doses Distributed to State**	39,630,080	9,500,250	19,018,995	25,514,685
**Doses Administered to State**	31,185,031	7, 281,555	15,980,527	19,205,116
**Percentage of Distributed Vaccines Administered**	78	76	84	75
**Percentage Fully Vaccinated**	32%	33%	36%	28%

Last Updated: 4 May 2021 * The seven-day average is the average of reported data between 28 June 2021 and 4 May 2021. The fourteen-day change is the moving average of reported data between 22 June 2021 and 4 May 2021.

**Table 2:** Dosing, schedule and ingredients of COVID-19 Vaccines Authorized for Emergency Use [92].

Vaccine	Dose	Dose Volume	Number of Doses/Series	Interval Between Doses	Active Ingredients	Inactive Ingredients
**Pfizer-BioNTech**	30 µg	0.3 mL	2	21 days	Nucleoside-modified mRNA encoding the viral spike (S) glycoprotein of SARS-CoV-2	2[(polyethylene glycol (PEG))-2000]-N,N-ditetradecylacetamide 1,2-distearoyl-sn-glycero-3-phosphocholine Cholesterol (4-hydroxybutyl) azanediyl) bis(hexane-6,1-diyl) bis(2-hexyldecanoate) Sodium chloride Monobasic potassium phosphate Potassium chloride Dibasic sodium phosphate dihydrate Sucrose
**Moderna**	100 µg	0.5 mL	2	28 days	Nucleoside-modified mRNA encoding the viral spike (S) glycoprotein of SARS-CoV-2	PEG2000-DMG: 1,2-dimyristoyl-rac-glycerol, methoxypolyethylene glycol 1,2-distearoyl-sn-glycero-3-phosphocholine Cholesterol SM-102: heptadecan-9-yl 8-((2-hydroxyethyl) (6-oxo-6-(undecyloxy) hexyl) amino) octanoate Tromethamine Tromethamine hydrochloride Acetic acid Sodium acetate Sucrose
**Johnson & Johnson**	5 × 10^10^ viral particles	0.5 mL	1	N/A	Recombinant, replication- incompetent Ad26 vector, encoding a stabilized variant of the SARS-CoV-2 Spike (S) protein	Polysorbate-80 2-hydroxypropyl-β- cyclodextrin Citric acid monohydrate Trisodium citrate dihydrate Sodium chloride Ethanol

**Table 3:** Pfizer-BioNTech COVID-19 Vaccine—Study Description [95].

Study Number	Phases	Description	BNT162b2— Number of Participants	Placebo— Number of Participants
**C4591001**	1, 2, and 3	Randomized, placebo-controlled, observer-blind. Evaluation: Efficacy, safety and immunogenicity.	Phase 1: 24 Phase 2/3: 21,823	Phase 1: 6 Phase 2/3: 21,828
**BNT162-01**	1 and 2	Randomized, open-label. Evaluation: Safety, immunogenicity and dose escalation.	Phase 1/2: 12	Phase 1/2: 0

**Table 4:** Moderna’s COVID-19 Vaccine—Study Description [97].

Study Number	Phases	Description	mRNA-1273— Number of Participants	Placebo— Number of Participants
**P301**	3	Randomized, placebo-controlled, observer-blind, stratified. Evaluation: Efficacy and safety.	Phase 3: 15,181	Phase 3: 15,170
**P201**	2	Randomized, placebo-controlled observer-blind, dose-confirmation. Evaluation: Immunogenicity and safety.		
**20-0003**	1	Open-label, dose-ranging. Evaluation: Immunogenicity and safety.		

**Table 5:** Janssen/Johnson & Johnson COVID-19 Vaccine—Study Description [100].

Study Number	Phases	Description	Ad26.COV2.S— Number of Participants	Placebo— Number of Participants
**3001**	3	Randomized, double-blind, placebo-controlled. Evaluation: Efficacy and safety.	Phase 3: 21,895	Phase 3: 21,888
**3009**	3	Randomized, double-blind, placebo-controlled. Evaluation: Efficacy and safety.		
**2001**	2a	Randomized, double-blind, placebo-controlled. Evaluation: Immunogenicity and safety.		
**1002**	1	Randomized, double-blind, placebo-controlled. Evaluation: Immunogenicity and safety.		
**1001**	1/2a	Randomized, double-blind, placebo-controlled. Evaluation: Immunogenicity and safety.		

**Table 6:** Vaccine candidates supported by BARDA and other federal agencies, reproduced from the Congressional Research Service (CRS) Insight IN11560 [108].

Company	Type	Contract Value	Specifications	Doses Per Person	Current Phase (Preliminary Effectiveness–U.S. Strain)	Storage
**Pfizer-** **BioNTech**	mRNA	$5.97B	300 million doses	2	Phase II/III (95%) EUA Issued	Ultra Cold Storage (−70 °C)
**Moderna**	mRNA	$4.94B $954M	300 million doses Development	2	Phase III (94.5%) EUA Issued	Cold Storage (6 mos, −20 °C) Refrigerator (30 days, −2 to −8 °C)
**Janssen/Johnson & Johnson**	Viral Vector	$1B $456M	100 million doses Development	1	Phase III (72%) EUA Issued	Refrigerator (3 mos, −2 to −8 °C)

**Table 7:** Federal governments contracts for ancillary COVID-19 vaccine supplies, reproduced from the Congressional Research Service (CRS) Insight IN11560 [108].

Company	Contract Value	Specifications
ApiJect Systems America	$138 million	100 million prefilled syringes by the end of 2020 Expansion of manufacturing capacity to produce 500 million prefilled syringes in 2021.
Corning Pharmaceutical Technologies	$204 million	Expansion of manufacturing capacity to produce an additional 164 million Valor Glass vials per year if needed.
SiO2 Materials Science	$143 million	Expansion of manufacturing capacity to produce 120 million glass-coated plastic containers per year if needed.
Becton, Dickinson and Co.	$42.3 million	Expansion of manufacturing capacity to produce needles and syringes.
Smiths Medical, Inc.	$20.6 million	Expansion of manufacturing capacity to produce needles and syringes.
Goldbelt Security, LLC	$125 million	530 million needles and syringes.
Retractable Technologies, Inc.	$53.6 million	Expansion of manufacturing capacity to produce safety needles and syringes.
Retractable Technologies, Inc.	$93.8 million	320 million needles and syringes.
Marathon Medical Corp.	$27.5 million	
Duopross Meditech Corporation	$48 million	134 million safety syringes by the end of 2020.
Cardinal Health Inc.	$15 million	500 million safety syringes over a 12-month period (August 2020–August 2021).
Gold Coast Medical Supply, LP	$14 million	
HTL STREFA Inc.	$12 million	
Quality Impact, Inc.	$9 million	
Medline Industries, Inc.	$6 million	

## Data Availability

Data sharing in this case report is not applicable as no new data were created or analyzed.
